# Breathing control training as a treatment for functional seizures (BREATHS trial): a multicentre, assessor-blinded, randomised controlled efficacy and acceptability trial study protocol

**DOI:** 10.1136/bmjopen-2025-107687

**Published:** 2026-01-30

**Authors:** Richard Kanaan, Rod Duncan, Cathrine Mihalopoulos, Sabine Braat, Terence J O’Brien, Dennis Velakoulis, Patrick Kwan, Roger T Mulder, Mark Cook, Saul Mullen, Deeanne Mayne, Gina Oliver, Dina Eleftheriadis, Ozayr Ameen, Mary Lou Chatterton, Wendyl D’Souza, John-Paul Nicolo, Piero Perucca, Toby Winton-Brown, Sophie Zaloumis, David Berlowitz

**Affiliations:** 1 Department of Psychiatry, Austin Health, The University of Melbourne, Melbourne, Victoria, Australia; 2 New Zealand Brain Research Institute, Christchurch, Canterbury, New Zealand; 3 School of Public Health and Preventive Medicine, Monash University, Clayton, Victoria, Australia; 4 Centre for Epidemiology and Biostatistics, University of Melbourne School of Population and Global Health, Carlton, Victoria, Australia; 5 MISCH (Methods and Implementation Support for Clinical Health) Research Hub, The University of Melbourne Faculty of Medicine Dentistry and Health Sciences, Melbourne, Victoria, Australia; 6 Department of Neuroscience, School of Translational Medicine, Monash University, Melbourne, Victoria, Australia; 7 Department of Neurology, The Royal Melbourne Hospital, Melbourne, Victoria, Australia; 8 Department of Neurology, Alfred Health, Melbourne, Victoria, Australia; 9 Psychological Medicine, University of Otago, Dunedin, New Zealand; 10 The University of Melbourne St Vincent’s Department of Medicine, Fitzroy, Victoria, Australia; 11 Department of Neurology, Bladin-Berkovic Comprehensive Epilepsy Program, Austin Health, Heidelberg, Victoria, Australia; 12 FND Hope Australia, Perth, Western Australia, Australia; 13 Department of Neurology, Christchurch Hospital, Christchurch, Canterbury, New Zealand; 14 University of Otago, Dunedin, Otago, New Zealand; 15 School of Public Health and Preventive Medicine, Monash University, Melbourne, Victoria, Australia; 16 Department of Medicine (Austin Health), The University of Melbourne, Melbourne, Victoria, Australia; 17 Alfred Mental Health and Addictions, Alfred Hospital, Melbourne, Victoria, Australia; 18 Centre for Epidemiology and Biostatistics, The University of Melbourne School of Population and Global Health, Melbourne, Victoria, Australia; 19 The University of Melbourne Department of Physiotherapy, Carlton, Victoria, Australia

**Keywords:** Functional Neurological Disorder, Seizures, Clinical Trial

## Abstract

**Introduction:**

Functional seizures (FS) are events that resemble epileptic seizures, but are not attributed to brain pathology and are instead thought to be due to psychological factors. A small, multisite, open-label, single-arm, pilot trial of a breathing intervention known as breathing control training (BCT) found it to be safe and effective in reducing seizure frequency in FS. We propose a protocol for a study to confirm these results.

**Methods and analysis:**

A 24-week, multicentre, individually-randomised, assessor-blinded, two-arm, parallel-group efficacy and acceptability trial of BCT versus control (Befriending) in 220 participants ≥16 years of age with FS. Eligible participants will be randomly allocated to receive two sessions of either BCT or Befriending over a 4-week period. Sessions will be delivered by a respiratory physiotherapist at a clinical care site or via telehealth. They will complete assessments prior to commencing treatment and at 4, 12 and 24 weeks after their initial session of BCT/Befriending. The trial will be conducted alongside treatment as usual. An economic evaluation including cost-utility and cost-effectiveness analyses will be carried out from health sector and societal perspectives.

**Ethics and dissemination:**

The study has been approved by The Austin Health Human Research Ethics Committee (HREC/84335/Austin-2022) and the New Zealand Central Health and Disability Ethics Committee (2022 FULL 12324). Findings will be reported to trial participants and consumers; presented at local, national and international conferences; and disseminated by a peer-reviewed scientific journal.

**STRENGTHS AND LIMITATIONS OF THIS STUDY**

- The study has a large sample, and an international, multicentre design.
- The study uses a control arm matching the intervention for therapist time, and delivered by the same therapist.
- The study is only blinded to assessor; participant and therapist blinding is not possible.

## Introduction

### Background and rationale

 Functional seizures (FS; also known as psychogenic or non-epileptic seizures) are events that resemble epileptic seizures, but are not attributed to brain pathology and are instead thought to be due to psychological factors.^1^ FS can occur many times per day, and their impact on patients and families is often disastrous, with most patients unable to work or study.^2^ FS affect all ages, with median onset in the 30s, and women three times more likely to be affected than men.^3^ People with FS are heavy users of a range of healthcare resources and are usually economically dependent.^4 5^ Studies show mortality is as increased in patients with FS as it is in those with epilepsy,^6^ and quality of life is significantly worse.^7^ It is common in neurological and epileptological practice, with an incidence of 7 per 100 000 per year, and a prevalence of approximately 0.05% of the general population.^8 9^ Despite this, treatment options for FS are limited.

The recommended treatment for FS is usually psychotherapy; however, a review of psychotherapy found no validated treatments offering sustained benefit.^10^ Psychological interventions are also resource demanding and expensive, and in many countries, limited by the availability of specialised psychologists and funding for them. Further, patient compliance is problematic and many patients do not attend or complete therapy,^11^ in large part because of the stigmatisation of psychiatric diagnoses.^12^ There is a need to develop a simpler and more acceptable treatment for patients with FS.

There is an association between FS and panic attacks, though the reason for this is unclear.^13^ We propose that the association is due to a common mechanism—hyperventilation. Symptoms of hyperventilation are commonly associated with FS,^13^ and FS can often be induced by asking patients to hyperventilate,^14^ suggesting a role for hyperventilation in the initiation of FS. We have confirmed this in our observations of 107 video electroencephalography (video EEG) recordings of patients with FS, finding a significant rise in respiratory rate in the minutes before the onset of seizures in most people.^15^

In patients with asthma, a therapy known as breathing control training (BCT) improves hyperventilation symptoms as shown on the Nijmegen scale of hyperventilation, anxiety and depression scores, and quality of life, but does not affect measures of airway function.^16^ We hypothesise that the same therapy when used for FS will improve breathing control, reduce hyperventilation and thus reduce the frequency of episodes. A standard course of BCT takes a single 1-hour session and a half-hour ‘refresher’ session at 1 month, administered by a physiotherapist. Its resource cost is substantially less than psychological interventions, which require several sessions, often more, by a specialised clinical psychologist. Even if BCT is effective in only a subset of patients, then it is likely to be very cost-effective, especially if we are able to determine which subset is likely to respond. BCT has the further advantage that it is likely to be acceptable to patients who may be deterred by psychological interventions, as it does not require that the patient frame or reframe their disorder as psychiatric.

We conducted a multisite, open-label, single-arm, pilot trial of BCT in FS.^17^ Participants with FS over the age of 16 years received an hour of breathing training from a respiratory physiotherapist, with a half-hour booster session a month later. Seizure frequency and Nijmegen scores (a measure of hyperventilation) were reported at baseline and follow-up, 3–4 months later. 18 subjects were recruited, and 10 completed follow-up. 7 of these 10 had improved seizure frequency, of which 3 achieved seizure remission, and 3 did not, with seizure frequency correlating with Nijmegen score (Spearman’s rank correlation=0.75). The intervention was well tolerated, with no adverse events (AEs) reported.

This provides preliminary data that BCT may be safe and effective for this patient group. This protocol is for a multicentre, randomised controlled trial to confirm these results.

### Study objectives

To establish the efficacy, acceptability and cost-effectiveness of BCT compared with Befriending in people ≥16 years of age with FS in a 24-week, multicentre, assessor-blinded, individually-randomised controlled superiority trial. In addition, for participants with routinely monitored transcutaneous carbon dioxide (tcCO_2_) during video EEG, we will examine if a respiratory biomarker of hyperventilation, tcCO_2_, will predict treatment response to BCT.

### Study hypothesis

We hypothesise that the proportion of patients with seizure remission, defined as FS frequency of zero in the preceding 4 weeks (recorded by the participant in a seizure frequency diary) at 12 weeks after initial treatment will be higher in the group of patients randomised to BCT compared with those randomised to control (Befriending).

Further, BCT will be as acceptable to patients and cost-effective compared with standard care for FS. In addition, we hypothesise that quality of life, work and social functioning, depression and anxiety symptoms, and hyperventilation tendency will improve more with BCT than control.

As an exploratory hypothesis, we expect hyperventilation preceding or during seizures will predict response to BCT, and that hyperventilation measured by expired carbon dioxide (CO_2_) will be clinically identifiable (by association with convulsive seizure phenomenology, childhood trauma history and increasing pre-ictal heart rate).

## Methods and analysis

### Trial design

A 24-week, multicentre, individually-randomised, assessor-blinded, two-arm, parallel-group efficacy and acceptability trial of BCT versus control (Befriending) in 220 participants ≥16 years of age with FS. Eligible participants will be randomly allocated (1:1) to receive two sessions of either BCT or Befriending with a respiratory physiotherapist over a 4-week period. They will complete assessments at baseline (prior to commencing treatment) and 4 weeks, 12 weeks and 24 weeks post their initial session of BCT/Befriending. The trial will be conducted alongside their standard care (treatment as usual). Optional, longitudinal observational follow-up will be conducted 52 weeks, 78 weeks and 104 weeks after their initial treatment session.

### Study setting

The trial will be conducted at four public hospitals in Melbourne, Australia (Austin Health, The Alfred, Royal Melbourne Hospital (RMH) and St Vincent’s Hospital Melbourne (SVHM)) as well as the New Zealand Brain Research Institute (NZ BRI) in Christchurch, New Zealand. Telehealth appointments will be used when in-person appointments are not feasible.

### Patient and public involvement

Patients were involved in the design and conduct of the trial. A representative of the consumer organisation for functional neurological disorders (FND), ‘FND Hope’, contributed to the design of the study and to obtaining funding, and sits on the trial steering committee.

### Intervention and comparator

#### Breathing control training

BCT includes teaching appropriate rate and depth of breathing and the development of a pattern of breathing appropriate to current physical activity. The elimination of dysfunctional breathing patterns, including hyperinflation and hyperventilation, is discussed. Diaphragmatic breathing is taught, and emphasis is placed on calm, slow, nasal expiration. Education on stress responses and their interactions with breathing patterns is given. The integration of appropriate breathing and relaxation techniques into daily living activities is encouraged. Initially, exercises are taught in a semi-recumbent position, progressing to sitting, then standing, then during everyday activities. Finally, the integration of breathing and relaxation techniques into speech is taught and practiced. Home exercises are given, with daily practice encouraged (5–10 min, two times a day) and recorded in a diary provided to participants.

The BCT will be delivered by a respiratory physiotherapist with specific training in this technique. They will conduct a 60-minute initial session with the participant (one-on-one) and a 30-minute refresher session 4 weeks later. The physiotherapist will administer the Brompton Breathing Pattern Assessment Tool (BPAT) in both sessions to assess for and monitor levels of breathing pattern irregularities.

#### Befriending (control)

The control condition, Befriending, is specifically designed to be the control intervention for clinical trials of psychology-based or ‘talking’ therapies. It offers the same clinician contact and involves meeting and talking with the participant in a supportive, friendly, but non-directive manner. It has been manualised and its outcomes formally assessed to allow it to serve as a theoretically justified ‘placebo’ group. It has become the standard control group in therapy trials for mental health, as it matches for expectancy, acceptability, enjoyment and engagement, while delivering no specific benefit on most outcomes.^18^ It will be administered by the same clinicians (physiotherapists) at intervals and duration matching the BCT sessions.

To provide equal opportunities, participants in the Befriending (control) group will be offered access to the BCT intervention (initial and booster session) after they have completed their 24-week follow-up questionnaires.

### Adherence

Adherence to BCT home exercises will be assessed from baseline to primary time point (Week 12). The physiotherapist will provide participants in the BCT group with a 3-month calendar style diary at the initial treatment session and ask them to record if and when they complete their recommended (daily) BCT exercises. The physiotherapist will review the adherence diary with the participant at the Week-4 booster session to gauge adherence to the treatment intervention. They will also contact the participant at Week 8 and Week 12 and ask them to return the adherence diary for these time points. Alternatively, BCT participants may complete the adherence diary via MyCap.

### Concomitant care

The BCT/Befriending will be used in addition to the participants’ standard care, that is, they will continue their ‘treatment as usual’ (TAU) while they are enrolled in the trial. Standard care can vary widely in this patient group, particularly when newly diagnosed with FS. For example, some patients may be taking (or withdrawing from) antiseizure medications and/or other pharmacotherapies; see a neuropsychiatrist, psychologist or other allied health professionals; whereas others may not be receiving treatment. There are no restrictions on what standard care can/cannot entail while they participate in the trial; however, if they are engaging in therapies which focus on specific breathing techniques (eg, box breathing, Buteyko method), they will be excluded from the trial.

Study staff will record all concomitant therapies at baseline and follow-up appointments, assisted by a tailored, self-reported Resource Use Questionnaire (RUQ), and for consenting Australian participants, accessing Medicare Benefits Schedule (MBS) and Pharmaceutical Benefits Scheme (PBS) data from Services Australia. For consenting Australian participants living in Victoria, health service use data (hospital admissions, emergency department presentations and community healthcare services) will also be retrieved from The Centre for Victorian Data Linkage (CVDL). Collectively, these data will capture what each participant’s TAU entails, as well as changes to TAU throughout the trial.

### Participants eligibility

Eligibility criteria are outlined in table 1.

**Table 1:** Inclusion and exclusion criteria

Inclusion criteria	Exclusion criteria
≥16 years of age	Comorbid epilepsy
Able to provide written, informed consent	Currently engaging in other breathing therapy treatments/clinical trials
Diagnosis of FS confirmed by an epileptologist based on at least one typical event captured on video EEG	
Self-reported FS frequency of at least two per month in the preceding 4 weeks	
English sufficient to complete questionnaires and understand the treatment intervention	

EEG, electroencephalography; FS, functional seizures.

### Participant safety

#### Risk management and safety

From our own pilot data, and randomised-controlled trials of breathing exercises for asthma, BCT’s excellent safety and tolerability profile is evident.^19 20^ Nonetheless, AEs will be reviewed and monitored throughout the trial to further explore BCT’s safety profile in FS specifically. As per the trial, physiotherapists (informed by their own clinical practice) expected side effects of BCT to include transient lightheadedness, breathlessness and/or feelings of panic in some individuals. These symptoms can be managed by the respiratory physiotherapist in the BCT session.

AEs will be defined as ‘any untoward medical occurrence in a…clinical trial participant administered a medicinal product and that does not necessarily have a causal relationship with this treatment’,^21^ and will include:

- Pre-existing conditions not reported at baseline.
- Worsening of a pre-existing condition.
- Diagnosis of a new condition/disease.
- New symptoms.
- Abnormal laboratory findings which are identified after randomisation into the trial

Serious AEs (SAEs) will be defined as ‘any adverse event that results in death, is life-threatening, requires hospitalisation or prolongation of existing hospitalisation, results in persistent or significant disability or incapacity, or is a congenital anomaly or birth defect’.^21^ In this trial, if a participant presents to emergency due to a FS, it will not be considered an SAE (as this can be a common occurrence in FS). However, details of the attendance will be reviewed and recorded by study staff, and also captured on the RUQ (self-reported by the participant).

The participant will be asked about the presence of adverse events at Week 4, 12 and 24, and details recorded in the corresponding Research Electronic Data Capture (REDCap) instrument. Severity, seriousness, relatedness (causality) and expectedness of AEs to the intervention will be assessed by the site principal investigator (PI) or delegate. All AEs/SAEs will be reported to an independent Data Safety Monitoring Board comprising an epileptologist, psychiatrist, physiotherapist and statistician for review. No interim analyses are planned.

After completion of the Childhood Experience of Care and Abuse Questionnaire (CECA-Q; self-report) at Week 12, participants will be encouraged to contact their study doctor if they have any concerns or become distressed. The contact details for their study doctor will be reiterated at the start of the questionnaire. For participants who disclose experiencing sexual abuse in childhood, a follow-up question will appear in REDCap asking if they would like the abuse to be reported to the authorities. Participants may select ‘Yes – I would like this to be reported. I understand that one of the study doctors will contact me to discuss the options available for reporting, including support services I can contact’ or ‘No – I do not give permission for this to be reported’. If they select ‘Yes’, one of the study doctors will contact the participant to discuss reporting procedures in further detail and address any of the participants queries/concerns. Contact details for support services will also be provided, if desired.

For participants who are eligible (FS onset within the last 2 years) and willing to undertake the Life Events and Difficulties Schedule (LEDS) at Week 12, there is a theoretical risk of distress as these assessments will explore potentially traumatic events from the participant’s past. In the event a participant should find these assessments distressing and which cannot be managed by the interviewer, they will be referred to neuropsychiatry clinics at their hospitals, free public clinics with specialist expertise in the diagnosis and management of FS and their complications; or escorted to the hospital’s emergency department if more urgent care is required.

### Outcomes

Primary, secondary and exploratory outcomes are outlined in table 2, box 1.

**Table 2:** Procedures in the trial that are in addition to standard care (alongside ‘treatment as usual’)

Procedure	Time/visit	Dosage/volume
BCT/Befriending sessions	Baseline and 4 weeks later	60 min initial session; 30 min second session
Seizure frequency diary	Completed by the participant as seizures occur throughout the 24-week study period	~1 min per week
Breathing exercises (BCT participants only) and adherence diary	Following the baseline session, through to Week 12	5–10 mins, two times a day
Self-report questionnaires	Baseline, Week 4, Week 12 and Week 24	30–45 mins
LEDS (optional)	Week 12	LEDS (optional) Week 12 ~2 hours

BCT, breathing control training; LEDS, Life Events and Difficulties Schedule.

Box 1 **Outcomes**

**Primary**

Seizure remission, defined as seizure frequency of zero in the preceding 4 weeks (recorded by the participant in a seizure frequency diary), measured at Week 12 (12 weeks post the initial treatment session).

**Secondary**

- Changes in hyperventilation from baseline, measured by the Nijmegen scale of hyperventilation provided at baseline, Week 4, Week 12 and Week 24.
- Changes in healthcare utilisation from baseline, measured via a tailored resource use questionnaire provided at baseline, Week 12 and Week 24, as well as changes in health service use ascertained from Services Australia and CVDL data (Services Australia and CVDL data for consenting participants only).
- Changes in quality of life from baseline, measured by the Assessment of Quality of Life Eight Dimension provided at baseline, Week 4, Week 12 and Week 24.
- Changes in depression and anxiety symptoms from baseline, measured by the 9-item Patient Health Questionnaire and Generalised Anxiety Disorder Assessment respectively, provided at baseline, Week 4, Week 12 and Week 24.
- Changes in occupational and social functioning from baseline, measured by the Work and Social Adjustment Scale provided at baseline, Week 4, Week 12 and Week 24.
- Acceptability of BCT captured by a tailored self-report questionnaire provided after attending the Week 4 ‘booster’ session.
- Safety of BCT assessed by the occurrence of adverse events reviewed at Week 4, Week 12 and Week 24.
- tcCO_2_ levels (mm Hg; a marker of hyperventilation) preceding or during seizures, BPAT score (at baseline) and/or Nijmegen score (at baseline), to predict response to treatment.

**Exploratory**

- The presence of significant life events in childhood and adolescence (≤16 years of age), and 12 months preceding the onset of their FS, assessed via the Childhood Experience of Care and Abuse Questionnaire and the LEDS*, respectively, to gauge moderators of treatment response.
- The MDI and Toronto Alexithymia Scale provided at baseline will be assessed if predictors of treatment response. MDI scores at Week 4, Week 12 and Week 24 will be assessed if they correlate with treatment response.

*Note, the LEDS is optional, and only relevant to participants whose FS have started within the last 2 years.BCT, breathing control training; BPAT, Brompton Breathing Pattern Assessment Tool; CVDL, Centre for Victorian Data Linkage; FS, functional seizures; LEDS, Life Events and Difficulties Schedule; MDI, Multiscale Dissociation Inventory; tcCO_2_, transcutaneous carbon dioxide.

### Sample size calculation and recruitment target

A total of 220 participants (110 per group) are required to detect a clinically relevant absolute risk difference of 20% or relative risk of 2 in the proportion of patients with FS remission between BCT (40%) and Befriending (20%) at Week 12 (80% power, 5% two-sided significance level, 25% drop-out). A remission rate of 14% with TAU has been demonstrated in our local population,^22^ but because Befriending may have some negligible effects, we have adopted a more conservative 20% remission rate for participants who are randomly allocated to the control arm. 40% FS remission at Week 12 (primary endpoint) in the active arm was chosen as the clinically significant aim of being twice the proportion typically achieving remission with current psychotherapies such as cognitive behavioural therapy.^22^ The dropout rate is a conservative estimate as we have achieved much lower with this population in other trials,^23^ and BCT has attrition of less than 10%.^24^

### Recruitment

Recruitment commenced in February 2024 is expected to remain open for a period of 4 years. Participants will be recruited from their clinical care site (Austin, Alfred, RMH, SVHM or Christchurch), either as an inpatient or outpatient, following their diagnosis of FS. Patients with FS who have previously received care at the hospitals (as an outpatient/inpatient) may also be contacted via letter from the site PI and followed up by telephone to gauge interest in study participation.

Following referral to the trial by their clinical care team (while a patient, or via study invitation letter), a member of the research team will contact the candidate to discuss the trial and address any of their queries. If willing, the candidate will be taken through a prescreening questionnaire to assess their eligibility for the trial (ascertaining age, FS diagnosis and frequency, epilepsy diagnosis, current treatments/therapies). If the criteria are satisfied, they will be provided a copy of the consent form and asked to review and then followed up by study staff at a time of their convenience. If willing to proceed with the trial, written, informed consent will be obtained and additional baseline data collected (participant’s medical history, including psychiatric history, presence of functional disorders and asthma). The participant will be asked to complete baseline self-report questionnaires via REDCap prior to randomisation into the trial (see ‘Allocation’ section below). As per hospital/government or health authority guidelines, the trial can be administered via Telehealth if needed, without significantly compromising efficacy of the intervention,^24 25^ and thereby limit potential disruptions to recruitment.

Recruitment from both inpatient and outpatient sources may lead to recruitment of a more chronic, and thus possibly more refractory, sample, but will increase the number of potential recruits and enhance generalisability.

### Allocation

#### Sequence generation

Participants will be individually randomised 1:1 to the BCT or the control arm (Befriending). A randomly permuted block randomisation list will be computer-generated by an independent statistician, stratified by site (ie, Austin, Alfred, RMH, SVHM or Christchurch) and hyperventilation symptoms (baseline Nijmegen score >23 vs ≤23).

#### Allocation concealment mechanism

Participants will be allocated sequentially following the randomisation schedule. The randomisation schedule will be stored by an independent statistician, as well as on REDCap, with access only permitted to the physiotherapists (who are unblinded during the trial).

#### Implementation

After prescreening and eligibility for the trial confirmed, and baseline self-report questionnaires completed (to obtain Nijmegen score for stratification purposes), the physiotherapist will contact the participant and inform them of their group allocation (randomisation) according to the randomisation allocation provided by REDCap.

### Blinding

The physiotherapists will deliver the intervention to participants in an unblinded manner. The research assistant (RA), who will be administering the outcome measures, as well as the statisticians involved in data analysis, will be blinded to treatment allocation while the trial is active. After randomisation, participants will be asked not to reveal their group allocation to the RA. Given many of the outcome measures will be administered electronically (via REDCap), this will also assist RA blinding.

### Participant safety and withdrawal

Participants will be withdrawn from the trial in any of the following circumstances:

1. At their own volition/withdrawal of consent.
2. Emergence of epileptic seizures.
3. Emergence of significant AEs, at request of the participant, or discretion of their clinician

If the participant wishes to withdraw, they will be asked to attend a brief telephone/in-person interview (the latter if convenient) to review their reason for withdrawal, the occurrence of AEs, as well as frequency of their FS (to gauge if the treatment has caused any worsening in clinical features). We will also ask them to sign the withdrawal of consent form to formally document their discontinuation and indicate if they wish for their data to be excluded from the analysis. We will, however, respect their wishes if they decline to partake in this exit interview.

If a participant self-reports to the study team (eg, during a routine study visit), or if a member of the study team identifies in the patient medical records a new diagnosis of epilepsy or the emergence of epileptic seizures, the participant will be withdrawn from the study. The diagnosis must be confirmed by an epileptologist. The participant will then be notified of their withdrawal from the trial and the reason for it. The standard withdrawal of consent procedure described above will be followed.

No withdrawn participants will be replaced.

### Data collection methods

#### Pre-screening

Following referral to the trial by their clinical care team, a member of the research team will contact the candidate to discuss the trial and address any of their queries. If willing, the candidate will be taken through a prescreening questionnaire to assess their eligibility for the trial (ascertaining age, FS diagnosis and frequency, epilepsy diagnosis, current treatments/therapies). If the criteria are satisfied, they will be provided a copy of the consent form and asked to review and then followed up by study staff at a time of their convenience. If willing to proceed with the trial, written, informed consent will be obtained and additional baseline data collected (participant’s medical history, including psychiatric history, presence of functional disorders and asthma).

#### Medical records

Participants will be asked to provide consent for study staff to access their medical record for purposes of the trial. The following will be extracted from their medical record where applicable/possible and data entered in a re-identifiable manner into an electronic Case Report Form hosted by REDCap:^26^

- FS frequency, features and semiology (convulsive/swoon/other, clinical hyperventilation symptoms).
- Age at onset of FS, age at diagnosis, duration of history, delay to diagnosis.
- Factors precipitating the onset of FS disorder.
- Psychiatric history.
  - Including anxiety/depression disorders, panic attacks/disorder, self-harm, trauma history including sexual/physical/emotional/marital abuse, psychiatric medication history, non-prescribed drug use, substance abuse/history of dissociation/somatization/health anxiety.
- Functional disorder history (including age at onset of functional disorders and details of diagnosis).
- Epilepsy history.
- Asthma history.
- tcCO_2_, EEG and MRI data.

#### Measures

In addition, the following measures and standardised clinical questionnaires will be used in the trial (see table 3, box 2, for scheduling). All self-reported questionnaires will be completed via REDCap.

**Table 3:** Participant timeline

Assessment/ procedure	Clinical assessment (neurologist)*	Prescreening	Initial session with physio (baseline)	‘Booster’ session (Week 4) with physio	Follow-up (Week 12†)	Follow-up (Week 24)	Week 52, 78 and 104 observational follow-up (optional)
Video EEG	x						
tcCO_2_ monitoring	x						
FS semiology and chronicity	x						
Medical hx (inc. family hx and psychiatric hx)	x						
Informed consent		x					
Confirm eligibility		x					
BCT/Befriending intervention			60 mins X	30 mins X			
Seizure frequency diary		For preceding 4 weeks	Recorded by the participant throughout the 24-week trial period (automated reminders sent weekly via text/email, or daily via MyCap)				For preceding 4 weeks
Seizure control and triggers		x			x		
Seizure quality		x		x	x		
Demographics		x					
AQoL-8D		x		x	x	x	x
Nijmegen scale		x		x	x	x	x
GAD-7		x		x	x	x	x
PHQ-9		x		x	x	x	x
WSAS		x		x	x	x	x
TAS		x					
MDI		x		x	x	x	x
RUQ		x			x	x	x
Adverse events				x	x	x	
Acceptability questionnaire				x			
LEDS (optional)					x		
CECA-Q					x		

* Standard care

† Primary endpoint

AQoL-8D, The Assessment of Quality of Life 8 Dimension; BCT, Breathing Control Training; CECA-Q, Childhood Experience of Care and Abuse Questionnaire; EEG, electroencephalogram; FS, functional seizures; GAD-7, Generalised Anxiety Disorder Assessment; Hx, history; LEDS, Life Events and Difficulties Schedule; MDI, Multiscale Dissociation Inventory; PHQ-9, Patient Health Questionnaire, 9-item; RUQ, Resource Use Questionnaire; TAS, Toronto Alexithymia Scale; WSAS, Work and Social Adjustment Scale.

Box 2 **Trial schematic**

**Clinical assessment (neurologist)**

- Functional seizures diagnosis confirmed by epileptologist.
- Participant informed of trial.
- Informed consent obtained OR obtain consent for study staff to follow-up and discuss.

**Prescreening**

- Eligibility confirmed using prescreening questionnaire.
- Informed consent (if not completed by referring clinician).
- Baseline study questionnaires completed: Demographics, The Assessment of Quality of Life Eight Dimension (AQoL-8D), Generalised Anxiety Disorder Assessment (GAD-7), Patient Health Questionnaire, 9-item (PHQ-9), Multiscale Dissociation Inventory (MDI), Nijmegen, Toronto Alexithymia Scale, Resource Use Questionnaire (RUQ), Seizure triggers and control, Seizure quality, Work and Social Adjustment Scale (WSAS).
- Medical record data extracted.

**Randomisation**

- Physiotherapist contacts participant to inform them of their group allocation and schedule first session.

**Initial treatment session (Breathing Control Training/Befriending): baseline**

- 60 min, one-on-one session with physiotherapist, conducted at clinical care site (or Telehealth if required).
- Seizure frequency and adherence diary provided.

**Booster treatment session (Breathing Control Training/Befriending): week 4**

- 30 min, one-on-one session with physiotherapist, conducted at clinical care site (or Telehealth if required).
- Adverse events, seizure frequency and adherence diary reviewed by physiotherapist.
- Research assistant (blinded) sends self-report questionnaires to participant via Research Electronic Data Capture (REDCap): *AQoL-8D, Acceptability, GAD-7, PHQ-9, MDI, Nijmegen, Seizure quality, WSAS.*

**Primary endpoint: week 12**

- Research assistant (blinded) sends participant self-report questionnaires via REDCap: *AQoL-8D, Childhood Experience of Care and Abuse Questionnaire, GAD-7, PHQ-9, MDI, Nijmegen, RUQ, Seizure triggers and control, Seizure quality, WSAS.*
- Adverse events and medication status reviewed by research assistant.
- For eligible and willing participants, invited to complete Life Events and Difficulties Schedule.

**Follow-up: week 24**

- Research assistant (blinded) sends participant self-report questionnaires via REDCap: *AQoL-8D, GAD-7, PHQ-9, MDI, Nijmegen, RUQ, Seizure quality, WSAS.*
- Participants randomised into the Befriending (control group) offered access to the BCT intervention.

**Observational follow-up: week 52, week 78 and week 104 (optional)**

- Participants sent self-report questionnaires via REDCap: *AQoL-8D, GAD-7, PHQ-9, MDI, Nijmegen, RUQ, WSAS, seizure frequency (for previous 4* *weeks).*

##### Acceptability

Acceptability of the intervention will be determined via a series of questions standardised during our pilot study. Examples include, ‘I found the training easy to understand…easy to do…easy to remember’ etc and the participant will be provided with a 5-point Likert scale ranging from ‘Strongly agree’ to ‘Strongly disagree’ for each question. We will also include a free-text section enabling the participant to provide additional feedback about the treatment if desired. This questionnaire is estimated to take between 2–5 mins to complete.

##### The Assessment of Quality of Life Eight Dimension

A 35-item, self-reported instrument which explores eight dimensions: Independent Living, Happiness, Mental Health, Coping, Relationships, Self-Worth, Pain and Senses (eg, vision, hearing). The participant is asked to select one response for each item based on their experiences from the past week. The Assessment of Quality of Life Eight Dimension (AQoL-8D) is estimated to take~5 mins to complete.^27^

##### Brompton Breathing Pattern Assessment Tool

A 7-item, respiratory clinician-administered instrument which can be used to assist in identifying dysfunctional breathing patterns. It requires the clinician to count the patient’s respiratory rate and observe their breathing pattern for 1 min before rating each item 0–2. Items include a rating of abdominal or upper chest movement, inspiratory flow, expiratory flow, channel of inspiration and expiration, air hunger, respiratory rate and rhythm. A total BPAT score ≥4 is indicative of breathing pattern disorders with a sensitivity of 92% and specificity of 75%.^28^

##### Childhood Experience of Care and Abuse Questionnaire

A self-reported version of the clinician administered childhood experience of care and abuse developed in 1994. The CECA-Q asks participants about relationships with their caregivers and other close relationships before they were 17 years of age. The CECA-Q is estimated to take between 20 and 30 min to complete.^29^

##### Generalised Anxiety Disorder Assessment

A 7-item, self-reported instrument which measures symptoms of anxiety based on the Diagnostic and Statistical Manual of Mental Disorders, Fourth Edition (DSM-IV) criteria for Generalised Anxiety Disorder (GAD). Responders are asked to rate how much each item applied to them in the past 2 weeks on a 3-point Likert scale (0=not at all; 3=nearly every day). Anxiety symptom severity is gauged by the instrument’s total score (0–4=None/minimal; 5–9=mild; 10–14=moderate; 15+=severe). The GAD-7 is estimated to take 1 min to complete.^30^

##### Life Events and Difficulties Schedule

A semi-structured interview designed to explore significant life events and related difficulties such as times of financial strain, unemployment, unstable housing, illnesses suffered by self or a family member and relationship difficulties; as well as important sources of social support for the interviewee. In this study, participants will be eligible to complete the LEDS if their FS have commenced within the preceding 2 years; however, it will be optional for eligible participants to complete. If they are willing, the RA will administer the LEDS at the Week 12 visit, and questions will be asked based on experiences within the 12 months prior to the onset of their FS. The administration time of the LEDS can vary; however, it usually takes between 2 and 3 hours and will be audio recorded to assist analysis. To improve reliability, interview details will be presented (in a de-identified manner) and reviewed by a panel who are blind to the participant’s subjective perceptions of explored events, as well as their health status at time of interview.^31^

##### Multiscale Dissociation Inventory

The Multiscale Dissociation Inventory (MDI) is a 30-item self-reported measure of dissociative symptomatology, which is considered a key contributing component in FS. The MDI consists of six different dissociative responses including disengagement, depersonalisation, derealisation, emotional constriction/numbing, memory disturbance and identity dissociation, which collectively provide a total ‘dissociation score’. It is estimated to take 10–15 min to complete.^32^

##### Nijmegen scale of hyperventilation

A 16 item, self-reported questionnaire which explores symptoms associated with dysfunctional breathing patterns, introduced over 35 years ago as a screening tool to detect patients with hyperventilation problems. Each item is answered by the participant on a 5-point Likert scale ranging from ‘never’ (equating to zero) to ‘very often’ (equating to four points). Total scores range from 0 to 64, with a score of over 19 indicating the presence of respiratory distress and dysfunction. The Nijmegen scale is estimated to take 2–3 minutes to complete.^33^

##### Patient Health Questionnaire, 9-item

A 9-item self-report questionnaire used to aid in the diagnosis of depression, as well as monitoring the severity of symptoms. Items correspond to the DSM-IV diagnostic criteria for major depressive disorder. Responders are asked to rate how much they have been bothered by each item based on the previous 2 weeks via a 3-point Likert scale (0=not at all; 3=nearly every day). An overall severity score is obtained by totaling the 9 items (mild depression: 5–9; moderate depression: 10–14; moderately severe depression: 15–19; severe depression: 20–27). The Patient Health Questionnaire, 9-item is estimated to take 1–2 min to complete.^34^

A tailored RUQ developed by health economists from Monash University (Melbourne, Australia) will ascertain participants’ utilisation of healthcare resources and lost productivity in the previous 3 months. This includes items which explore medication use, emergency department presentations, ambulance assistance, hospitalisations and clinician visits. The RUQ is expected to take 5–8 mins to complete.

Participants randomised into the BCT group will be provided with a second diary to record if and when they complete their BCT home exercises up until primary endpoint (Week 12). This diary will also be available to complete on MyCap. If the latter, participants will receive a daily notification on their smartphone/tablet device asking them ‘did you complete your BCT homework yesterday?’ (yes/no).

Participants completing pen and paper diaries (or electronically on a computer) will be reminded by the physiotherapist to return the first month’s diary at the ‘booster’ session for review. Thereafter, at the end of each monthly cycle until Week 24, participants will be contacted by study staff (emailed/texted) and asked to return their seizure diary (via email/post as they prefer). If participants do not respond to the email/text, they will be telephoned by study staff within 5 days to assist gathering this data. A copy of the diary will be uploaded into REDCap and FS frequency for the preceding 4 weeks calculated.

##### Seizure Control and Triggers Questionnaire

A 19-item clinical questionnaire we developed (and used in the pilot study) which asks the participant to specify symptoms which precede their FS (eg, feeling scared, short of breath, lightheaded, tingling sensations) as well as their perceived control over the attacks (‘I can delay having a seizure if I need to… I can stop myself having a seizure…’). Participants may select one response per item from the following: never, sometimes, usually, always. It is estimated to take between 2 and 3 min to complete.

##### Seizure Quality Questionnaire

A simple 3-item questionnaire adapted from the 14-item ‘Seizure Severity Questionnaire’. Participants are asked how severe (intense), as well as how bothersome (interfering) their FS were (overall) in the past 4 weeks on a Visual Analogue Scale from 1 to 7. The third item asks if the duration of their FS has reduced in the previous 4 weeks using a 5-point Likert scale (strongly agree to strongly disagree). This questionnaire is anticipated to take 30–60 s to complete.

##### Toronto Alexithymia Scale

A 20-item, self-reported questionnaire and one of the most commonly used to measure alexithymia (difficulties a person may have in identifying and describing emotions). Each item is rated on a 5-point Likert scale ranging from ‘Strongly agree’ to ‘Strongly disagree’, with higher scores indicating greater difficulties. It is estimated to take 5 min to complete.^35^

##### Work and Social Adjustment Scale

A brief, 5-item, self-reported measure which captures the participant’s views regarding functioning in terms of work, home management, social leisure and private leisure activities, family and other relationships. The WSAS is estimated to take 2–3 min to complete.^36^

### Observational follow-up

52 weeks, 78 weeks and 104 weeks after their initial treatment session, participants will be sent online questionnaires (AQoL-8D, MDI, Nijmegen scale, FS frequency for the preceding 4 weeks, RUQ, WSAS) to complete. These longitudinal follow-up questionnaires will be optional, with the participant being able to opt out at any stage. These data will allow the researchers to observe FS health outcomes over an extended period of time and explore if there are any long-term benefits of BCT for FS.

### Health service use databanks

For consenting Australian participants, data will be requested from Services Australia to ascertain use of MBS and PBS services. For consenting Victorian participants, data regarding hospital admissions, emergency department presentations and community health service use will be retrieved from CVDL. Both Services Australia and CVDL data will be requested for the period of study participation (ie, the 24-week randomised, controlled period), as well as 3 months prior to randomisation and will assist the health economic evaluation of the project and determining each participant’s TAU.

### Training and fidelity

Prior to participant recruitment and data collection, a 5-day intersite meeting will be conducted to train the physiotherapists assigned to provide the interventions (BCT/Befriending) and obtain inter-rater reliability statistics. Fidelity will be assessed by a self-administered checklist completed by the physiotherapist at the end of each BCT/Befriending session, and audio recording a random sample (5%) of the treatment sessions. Regarding the LEDS, inter-rater reliability will be assessed before commencement of the trial and repeated every 6 months, using a standardised taped patient interview. Raters will need to achieve an intraclass correlation of 0.8 and will undergo further training as necessary to achieve it.

### Record retention and storage

Data collected and entered into REDCap will be stored centrally on REDCap servers facilitated by The University of Melbourne. All hard copy data (eg, signed consent forms, LEDS assessments) will be stored at the site where the participant has been consented (ie, Austin, Alfred, RMH, SVHM or Christchurch) in a locked filing cabinet accessible only to approved study staff. Hard copy and electronic data will be stored for a minimum of 15 years post study closure and then destroyed under guidance and supervision of the Sponsor’s record management policies.

### Statistical analysis

A formal detailed statistical analysis plan for the study will be written prior to unblinding of the database. All analyses will follow the intention-to-treat principle.

#### Primary outcome

FS remission (table 2) will be analysed using a random intercept logistic regression model, including in the model factors visit, study group, visit by study group interaction and the stratification factors. This model will be further adjusted for FS frequency at baseline. The relative and absolute treatment effect estimate and corresponding two-sided 95% CI, and p value of BCT compared with Befriending at Week 12 will be obtained to evaluate the primary hypothesis. In addition, an adjusted treatment effect will be obtained by including potential covariate (eg, depression) in the model.

#### Secondary outcomes

Binary outcomes will be analysed similarly to the primary outcome; continuous outcomes will be analysed using a Longitudinal Data Analysis model.^37^ Any markedly skewed continuous outcomes will be transformed before analysis. The acceptability of BCT captured by a tailored self-report questionnaire provided after attending the Week 4 ‘booster’ session and adverse events will be reported by study group and visit.

The statistical models for the primary and secondary outcomes will provide valid inference in the presence of missing data if the missing data mechanism is missing at random.

#### Economic evaluation

Cost-utility and cost-effectiveness analyses will be carried out from health sector and societal perspectives. The health sector perspective includes the cost to provide BCT and the cost of concurrent healthcare service use. Detailed costing of the BCT intervention will be performed using micro-costing methods. The number and types of additional health services used by participants over the period of the trial will be collected with a resource use questionnaire. As most study participants will be recruited in Australia, a one-country costing approach will be applied, using unit cost estimates from Australia.^38^

The societal perspective will add the cost of lost productivity (absenteeism and presenteeism) to the health sector costs. Lost productivity will be measured with questions in the resource use questionnaire and valued using the human capital method applying an average Australian wage rate plus oncosts to time missed from work.

The AQoL-8D utility values for each participant at each time point will be used to calculate quality-adjusted life-years (QALYs) using the area under the curve method. The within-trial economic evaluation will calculate an incremental cost-utility ratio as the difference in total costs between the BCT and control groups divided by the difference in QALYs between groups. Seizure remission (primary outcome) will be used to calculate an incremental cost-effectiveness ratio as the difference in total costs between the groups divided by the difference in remission rates. Non-parametric bootstrapping will be used to determine CIs for the incremental cost-utility and cost-effectiveness ratios. Acceptability curves will also be constructed to determine the probability of cost-effectiveness against the commonly used Australian willingness to pay threshold of $A50 000/QALY.

Missing data will be explored and managed for the resource use and AQoL-8D questionnaires based on recommendations for analysis of trial-based economic evaluations with missing data.^39^ Sensitivity analyses will be undertaken to evaluate the robustness of results when using administrative cost data from MBS/PBS and CVDL as well as changes to analytical assumptions (IE, management of missing data).

## Ethics and dissemination

### Research ethics approval

The project has been approved by The Austin Health Human Research Ethics Committee (HREC/84335/Austin-2022) under the National Health and Medical Research Council (NHMRC) developed National Certification Scheme of Institutional Processes Related to the Ethical Review of Multi-centre Research (National Certification Scheme). Once approval was granted for the four Melbourne sites, the project was reviewed by the New Zealand Central Health and Disability Ethics Committee (2022 FULL 12324).

### Consent

Participants may be consented into the trial by the referring clinician, the RA or the physiotherapist, who will follow the standard operating procedure for informed consent specific to the participant’s clinical care site. All participants will need to provide written informed consent prior to commencing the study and data collection occurring (excluding data obtained on the prescreening questionnaire used to determine eligibility). Participants may consent via (1) a hard-copy, site-specific consent form (see online supplemental material) or (2) electronically via REDCap. The latter will allow participants who are discharged from their clinical care site to consent to the study from home at their convenience. Participants below the age of 18 will be required to discuss the trial with their parent/guardian and both the young person and parent/guardian will be required to co-sign the consent form. After first receiving the consent form, the candidate may have as long as they wish to consider proceeding with the trial (while recruitment for the project is open).

For Australian participants, consent will be sought to access data on use of healthcare services through MBS and PBS. A specific consent form, following guidelines from Services Australia, will be provided for this (optional) component. In addition, Victorian participants will be asked to provide consent on their site-specific consent form to enable CVDL data retrieval. Participants will also be asked to provide extended consent for their non-identifiable data to be used in future projects closely related to this project, or in the same general area (as per the NHMRC National Statement, 2018). If significant changes to the study design occur, all participants will be contacted and asked to review an amended consent form outlining these changes, allowing them to re-negotiate their consent.

### Confidentiality

Study staff at each site will maintain password protected potential participant, and a randomised participant spreadsheet which will contain participant contact details, and for randomised participants, details of their emergency contact/s and their treating team. These spreadsheets will also link the participant’s designated alpha and numeric participant code with their personal information to enable re-identification of collected study data (ie, self-reported questionnaires and other data contained in REDCap). These spreadsheets will be stored on local hospital servers and require study staff log-in credentials to access (username and password). Signed participant information and consent forms will be kept in locked filing cabinets at the participant’s clinical care site (Austin Health, The Alfred, RMH, SVHM or NZ BRI).

### Dissemination policy

The primary manuscript, detailing the study methodology and results, will be submitted to a peer-reviewed scientific journal to be considered for publication. The results of the project will be communicated to trial participants if desired via an HREC approved letter outlining a lay summary of the results, as well as with the consumer organisation for FND, ‘FND Hope’.

## Discussion

This study described the design and methodology of the first multicentre, randomised, assessor-blinded, two-arm, parallel-group efficacy and acceptability trial of BCT versus control in patients with FS. Despite increased research in interventions for FS, and an abundance of smaller studies suggestive of therapeutic effect, there remain no definitive, validated treatments.^10^ Once completed, this study will represent only the second appropriately powered, multicentre, randomised trial of an intervention for FS.^22^ We hypothesise that BCT will reduce the seizures and related symptoms of FS greater than a control intervention designed to provide no benefit.

BCT has been found to be safe and well-tolerated when provided to people with respiratory conditions.^25^ As a brief intervention, it should prove cheap to deliver, and as an allied health intervention, it may prove more acceptable than psychotherapy interventions, where large proportions will not participate or complete the intervention.^22^ Its efficacy in FS remains to be determined, as it has not been subject to a trial other than our own pilot.^17^

The mechanism of any effect remains to be determined, but the therapy was designed to address abnormal breathing patterns, particularly hyperventilation, and in our pilot study, improvement correlated with improvement in this. People with FS do commonly have abnormal breathing, not only around the time of their FS.^13^ There are a priori reasons to think the intervention may work in FS, in the same way that it has worked in asthma. A goal of this study is to explore the mechanism of any effect, particularly in regard to breathing pattern, whether measured by clinical observation of breathing patterns, questionnaires or CO_2_ measurements. If the intervention is only effective in a subgroup defined by its breathing pattern, we would hope to be able to identify it.

The large sample size and randomised placebo-controlled design are significant methodological strengths in the current study. A further strength is the assessment of clinical outcomes at prolonged follow-up. Previous clinical trials have produced impressive results at the end of the intervention, only for a substantial proportion to relapse in follow-up.^10^ A final advantage for this study is the heterogeneity of the sample. The broad inclusion and limited exclusion criteria mean that participants experiencing a range of psychiatric comorbidities will be eligible.

## Supplementary material

10.1136/bmjopen-2025-107687 online supplemental file 1
