# Supplementary material for: Breathing control training as a treatment for functional seizures (BREATHS trial): a multicentre, assessor-blinded, randomised controlled efficacy and acceptability trial study protocol
Source: BMJ Open. 2026 Jan 30;16(1):e107687. doi: 10.1136/bmjopen-2025-107687 (PMC12863316; doi:10.1136/bmjopen-2025-107687)
Supplement: online supplemental file 1 [file bmjopen-16-1-s001.pdf]

Participant Information Sheet/Consent Form

Interventional Study - *Adult providing own consent*

[Insert site name]

**Title**	Breathing Control Training (BCT) as a Treatment for Functional Seizures (FS)
**Short Title**	BREATHS trial
**Protocol Number (Version)**	1 (5)
**Project Sponsor**	The University of Melbourne
**Coordinating Principal Investigator/ Principal Investigator**	Professor Richard Kanaan
**Associate Investigator(s)** *(if required by institution)*	*[Associate Investigator(s)]*
**Location** *(where CPI/PI will recruit)*	*[Name of Institution]*

Part 1 What does my participation involve?

1 Introduction

You are invited to take part in this research project. This is because you experience Functional Seizures (or 'FS' for short). The research project is testing a new treatment option for FS. The new treatment is a breathing technique called 'Breathing Control Training' (or BCT for short).

This Participant Information Sheet/Consent Form tells you about the research project. It explains the tests and treatments involved. Knowing what is involved will help you decide if you want to take part in the research.

Please read this information carefully. Ask questions about anything that you don't understand or want to know more about. Before deciding whether or not to take part, you might want to talk about it with a relative, friend or your local doctor.

Participation in this research project is voluntary. If you don't wish to take part, you don't have to. You will still be able to access care whether or not you take part.

If you decide you want to take part in the research project, you will be asked to sign the consent section. By signing it you are telling us that you:
- Understand what you have read
- Consent to take part in the research project
- Consent to have the tests and treatments that are described
- Consent to the use of your personal and health information as described

You will be given a copy of this Participant Information and Consent Form to keep.

2 What is the purpose of this research?

We do not understand very clearly what causes Functional Seizures (FS) or how to treat them. Previous research suggests that rapid breathing (called hyperventilating) can provoke an episode in some people who have FS. We think that if these people could learn to control their breathing so that they didn't breathe too quickly this might stop them having seizures. We have tested this on a few people with FS by teaching them something called *Breathing Control Training* (or 'BCT' for short) and found that most of their seizures either stopped or became much less frequent. BCT is a treatment provided by physiotherapists for people with asthma so they can manage their hyperventilation symptoms, but we now want to see if it works for FS.

In this research project, we will provide BCT to a larger group of people with FS to find out how many people it works for, and to try and figure out which ones it does help (so we know who to recommend the treatment to in future). We will also assess if BCT is safe for FS (i.e. does it cause any side effects); if it is acceptable (i.e. do people like it and find it easy to implement); and if it is an affordable treatment option for people with FS.

BCT is an experimental treatment for FS. This means that it is not an approved treatment for FS in Australia, and therefore, should be tested scientifically to see if it is an effective treatment option.

3 What does participation in this research involve?

If you would like to take part in this research project, you will be given this information document and asked to read through carefully. If you have any questions or queries, please reach out to a member from the research team who can discuss your concerns with you. You must sign the consent page before any of the research project assessments and treatments commence.

Firstly, you will meet with a member of the research team who will ask you some questions to confirm you are eligible to take part in this project. This will include some questions about your FS symptoms, diagnosis and treatments, as well as how old you are. They may also collect some information that is stored in your medical record at your clinical care site, for example, they may collect details about your FS diagnosis and related test results, as well as other medical conditions you have. This extracted information would only be used for purposes of this research project so we can better understand the participants that we recruit. We hope that accessing this information will make it less burdensome for you when trying to remember specific details about your medical history.

If you are eligible and willing to proceed with the project, you will be randomly allocated into one of two groups: 1) the group of participants who receive the BCT treatment or 2) the 'Befriending' group. If you are allocated into the BCT group, a physiotherapist will teach you how to control and calm your breathing. If you are allocated into the Befriending group, you will meet with a member of the research team and chat about topics which you find interesting. For example, it might be discussing hobbies you enjoy, your favourite books, a movie you saw recently, or holidays you've had in the past.

Before you commence the BCT or Befriending, you will be sent some online questionnaires to complete which will ask you about your demographic and health information (for example, date of birth, gender, postcode, education, employment status, smoking status); symptoms related to your breathing; depression and anxiety symptoms; quality of life; your social, recreational and work functioning; as well as different healthcare services you use (for example, medical appointments you attend). We anticipate these questionnaires may take between 30-40 minutes to complete.

You will then meet with a physiotherapist who will provide your first session of BCT or Befriending (60 minutes). This will take place at your clinical care site, or via Telehealth if required. If you are in the BCT group, you will be encouraged to practice the techniques you learn from the

physiotherapist twice a day, for about 5-10 minutes at a time. Four weeks after your first treatment session, we will also provide a 'booster' session (approx. 30 minutes) where you will meet with the physiotherapist again to review your progress and offer additional training if you need (or you will have another Befriending session for 30 minutes if this is the group you are enrolled in). In some cases, your sessions may be audio recorded so the researchers can make sure that the physiotherapists are delivering the treatments accurately. The physiotherapist will inform you if your session is going to be audio recorded before they start the recording device.

Whilst you are participating in the study, we ask that you complete a diary to record how often you are having seizures over a 6-month period. You can complete the diary via a mobile phone app, or via a pen & paper diary, as you prefer. If you'd like to use the app, you'll receive a daily notification on your phone reminding you to complete the diary. If you prefer pen & paper, we will send you an email or text once a week reminding you to complete the diary. In addition, a member of the research team will contact you at the end of each month and ask you to return the pen & paper diary for the previous month.

There will also be some additional questionnaires, similar to those that you completed prior to starting the treatment, which will be sent to you via email 4-weeks after starting the treatment (coinciding with the booster session), 12-weeks, and also 24-weeks after starting the treatment. The follow-up questionnaires will give you a chance to provide feedback about the treatment if desired. At Week-12, there is an additional questionnaire which will ask you about experiences from your childhood. If you disclose that you experienced sexual abuse in childhood, you will be given the option for this to be reported to the authorities, with your permission.

Optional: For participants who started experiencing seizures within the last 2-years, you can complete an additional interview-style questionnaire at Week-12. This would involve meeting with a member of the research team who will ask you about different life events you may have encountered within the 12-months prior to having seizures. This interview can take up to two hours and will be audio recorded. It is expected that the data collected from this questionnaire will form part of a PhD at the University of Melbourne for *[student name]*.

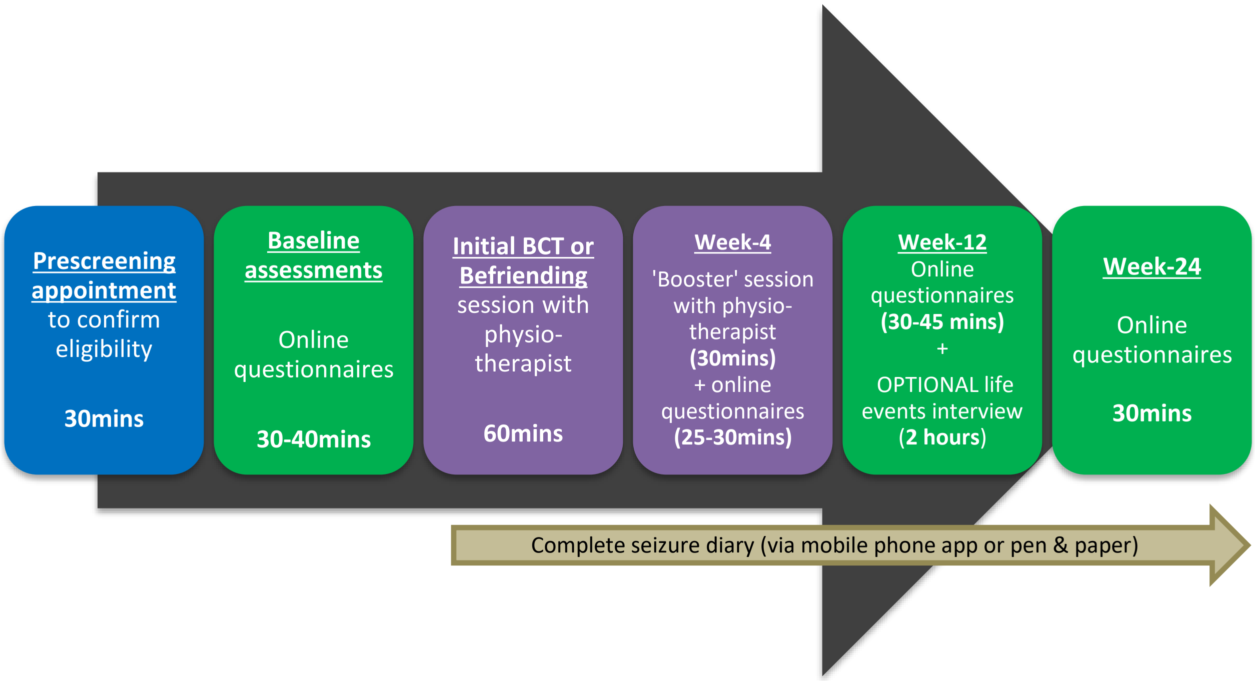

Figure 1. Flowchart of what participation involves in this research project

The researchers would also like to review people's progress over an extended period of time so they can assess if there are any long-term benefits of the BCT. This would involve completing some additional online questionnaires emailed to you approximately 6-months, 12-months and 18-months after you complete the study. We expect the questionnaires will take approximately 30 minutes to complete each time. If you do not wish to complete these questionnaires, you may

simply ignore them; you are also welcome to opt out at any time, in which case we will not send them to you.

You will be participating in a randomised controlled research project. Sometimes we do not know which treatment is best for treating a condition. To find out we need to compare different treatments. We put people into groups and give each group a different treatment (in this research project, the BCT or Befriending). The results are compared to see if one is better. To try to make sure the groups are the same, each participant is put into a group by chance (random). In this project, you will be randomly allocated into one of two groups, the BCT group, or the Befriending group, with an equal chance of being allocated into either.

It is desirable that your local doctor be advised of your decision to participate in this research project. With your permission, we can send a letter to your doctor, and other health care professional/s of your choice, to let them know you have been enrolled into the project.

There are no additional costs associated with participating in this research project, nor will you be paid. All treatments offered in the research project will be provided to you free of charge. You may be reimbursed for any reasonable travel, parking, meals and other expenses associated with attending the research project visits (up to $50 AUD per visit).

Optional: With your permission, the researchers would also like to collect data regarding your health service use from the Medicare Benefits Schedule (MBS) and the Pharmaceutical Benefits Scheme (PBS) via Services Australia. This is so we can see how many doctors' appointments you attend, which medications you take, and how much these services cost you. This will help us assess how much you spend to have your FS treated, and also if it costs you less as you participate in this research project. We will also use this data to assess if the treatment we are providing (BCT) is more cost effective than standard treatments for FS.

Services Australia is not involved in this research project other than to provide the information that you have consented to the release of, should you decide to participate in this study. Services Australia has confirmed that this research and any associated documents have received approval from a Human Research Ethics Committee (HREC) that is registered with and operates within guidelines set out by the National Health and Medical Research Council (NHMRC). You will be asked to sign a separate consent form authorising the study to access your Services Australia information, see the separate Services Australia Participant Information Document and Participant Consent Form.

Optional: To assist us further in analysing the cost-effectiveness of BCT, we would like your permission to retrieve information on your hospital and emergency department admissions, as well as community and mental health service use through The Centre for Victorian Data Linkage (CVDL). This will include the total number of times you have used any of these services in the 3-month period prior to starting the study, and whilst you are participating. Please indicate on page 10 of this document if you permit the researchers to access this data.

4 What do I have to do?

Taking part in this project does not require you to change any other aspect of your lifestyle or medical treatment. Specifically, you may still have any other treatments for your FS that you or your doctors arrange. If you commence any new treatments or therapies, for example, a new medication, please let a member from the research project know. We will also ask you about treatments and other therapies you use in the online questionnaires you complete throughout the research project.

One thing that would exclude you from taking part in the study is if you are currently engaging in other breathing therapies. This is because it makes it harder for us to assess if BCT will work or not. A member of the research team will discuss if this is relevant to you prior to commencing this project.

5 Other relevant information about the research project

This research project has been funded by the National Health and Medical Research Council (NHMRC).

The researchers are planning to enrol 220 people into this project over a 4-year period. We will enrol participants in Australia and New Zealand at 1. Austin Health (Heidelberg, VIC), 2. The Alfred Hospital (Prahran, VIC), 3. The Royal Melbourne Hospital (Parkville, VIC), 4. St Vincent's Hospital (East Melbourne, VIC) and 5. The New Zealand Brain Research Institute (Christchurch, New Zealand). The project involves researchers from The University of Melbourne, Monash University, University of Otago, and a representative from FND Hope, a global charity and support network for people with Functional Neurological Disorder.

6 Do I have to take part in this research project?

Participation in any research project is voluntary. If you do not wish to take part, you do not have to. If you decide to take part and later change your mind, you are free to withdraw from the project at any stage.

If you do decide to take part, you will be given this Participant Information and Consent Form to sign and you will be given a copy to keep.

Your decision whether to take part or not to take part, or to take part and then withdraw, will not affect your routine treatment, your relationship with those treating you or your relationship with *[clinical care site].*

7 What are the alternatives to participation?

You do not have to take part in this research project to receive treatment at this hospital, and your decision to take part, or not take part, will not affect the care you receive. Your study doctor can discuss treatment options for FS with you, which may include referral to a neuropsychiatrist if you desire. Your participation in this project will not affect that.

8 What are the possible benefits of taking part?

We cannot guarantee or promise that you will receive any benefits from this research. However, there is a chance that your seizures may improve. By participating in this research project, you may help us discover if BCT is an effective treatment for other people living with FS.

9 What are the possible risks and disadvantages of taking part?

There are no known side effects of BCT (or Befriending). However, given this is one of the first times BCT has been researched in a large group of people with FS, there may be side effects that the researchers do not expect or do not know about and that may be serious. Tell your study doctor immediately about any new or unusual symptoms that you get.

Many side effects go away shortly after treatment ends. However, sometimes side effects can be serious, long lasting or permanent. If a severe side effect or reaction occurs, your study doctor may need to stop your treatment. Your study doctor will discuss the best way of managing any side effects with you and arrange for you to have them treated if required.

For those participants who partake in the optional life-event questionnaire at Week-12, it is possible you may find answering some of the questions upsetting. If so, one of the study doctors will arrange for you to see a psychiatrist or psychologist who understands FS, if desired. Any support will be provided by qualified staff and will be provided free of charge.

10 What if new information arises during this research project?

Sometimes during the course of a research project, new information becomes available about the treatment that is being studied. If this happens, your study doctor will tell you about it and discuss with you whether you want to continue in the research project. If you decide to withdraw, your study doctor will make arrangements for your regular health care to continue. If you decide to continue in the research project you will be asked to sign an updated consent form.

Also, on receiving new information, your study doctor might consider it to be in your best interests to withdraw you from the research project. If this happens, they will explain the reasons and arrange for your regular health care to continue.

11 Can I have other treatments during this research project?

Yes. Although please tell your study team about any changes to your treatment during your participation in the research project, including prescription and over-the-counter medications, psychology, vitamins or herbal remedies, acupuncture or other alternative treatments.

12 What if I withdraw from this research project?

If you decide to withdraw from the project, please notify a member of the research team before you withdraw. This notice will allow that person or the research supervisor to discuss any health risks or special requirements linked to withdrawing.

If you do withdraw your consent during the research project, the study doctor and relevant study staff will not collect additional personal information from you, although personal information already collected will be retained to ensure that the results of the research project can be measured properly and to comply with law. You should be aware that data collected up to the time you withdraw will form part of the research project results. If you do not want them to do this, please let the study staff know when you withdraw by specifying on the 'Withdrawal of Participation' form you will be provided.

13 Could this research project be stopped unexpectedly?

This research project may be stopped unexpectedly for a variety of reasons. These may include reasons such as:

- Unacceptable side effects
- The treatment being shown not to be effective
- The treatment being shown to work and not needing further testing
- Decisions made by local regulatory/health authorities

14 What happens when the research project ends?

For those participants who were allocated to the Befriending group, we will offer you the BCT treatment after you have completed the Week-24 follow-up questionnaires. This will be the same BCT as though you had been allocated to it in the first place, the only difference being that you will not have to complete the questionnaires again.

Please let a member of the research team know if you would like to be informed of the study results, and we will let you know in the manner you choose. Please note, the results of the study will be available after recruitment and data collection at all hospital sites has finished, data analysis performed, and the findings published. This may not be until after 2026.

Part 2 How is the research project being conducted?

15 What will happen to information about me?

By signing the consent form, you consent to the study doctor and relevant research staff collecting and using personal information about you for this research project. Any information obtained in connection with this research project that can identify you will remain confidential. We will use an alpha and numeric (participant) code to identify your data, and only the researchers and staff members involved in the project will be able to link up your personal details with your designated participant code. Any data that does have your identifying details will be stored on password protected spreadsheets (if electronic files), or stored in locked filing cabinets (if paper copies) at the hospital where you receive your clinical care. Access to this information will only be permitted to approved study staff. Your information will only be used for the purpose of this research project and it will only be disclosed with your permission, except as required by law.

Information about you may be obtained from your health records held at this and other health services for the purpose of this research. By signing the consent form, you agree to the study team accessing health records if they are relevant to your participation in this research project. Information about your participation in this research project may be recorded in the medical record held at your clinical care site.

Information obtained during the research project is subject to inspection (for the purpose of verifying the procedures and the data) by the relevant authorities and authorised representatives of the Sponsor, The University of Melbourne, the institution relevant to this Participant Information Sheet, *[Name of institution]*, or as required by law. By signing the Consent Form, you authorise release of, or access to, this confidential information to the relevant study personnel and regulatory authorities as noted above.

The NHMRC strongly encourages data sharing between researchers who may also be investigating the same health condition or related aspects. This allows researchers to collaborate and ultimately increase knowledge about the condition in question – often in less time than if they were working independently. In this study, you have the option to provide *'extended consent'* which means data collected from you may be used for future research projects related to this one, or a similar area. It is important to note that any data that is shared will be non-identifiable, meaning all of your identifying details will be removed, unless you specifically give permission otherwise, and it could only be contributed to projects which have been reviewed and approved by a Human Research Ethics Committee (HREC).

It is anticipated that the results of this research project will be published and/or presented in a variety of forums. In any publication and/or presentation, information will be provided in such a way that you cannot be identified, except with your permission. Once the research project has concluded, and the study results finalised, the data that we collected from you will be stored securely for a period of 15 years, and then it will be destroyed under the supervision of the project Sponsor, The University of Melbourne.

In accordance with relevant Australian and/or Victorian privacy and other relevant laws, you have the right to request access to your information collected and stored by the research team. You also have the right to request that any information with which you disagree be corrected. Please contact a member from the research project (named at the end of this document) if you would like to access your information.

16 Complaints and compensation

If you have any complaints about any aspect of this research project, including the way it is being conducted, please contact the following who can assist you through this process:

Name	*Local Complaints Officer*
Telephone	
Email	

If you suffer any injuries or complications as a result of this research project, you should contact the study team as soon as possible and you will be assisted with arranging appropriate medical treatment. If you are eligible for Medicare, you can receive any medical treatment required to treat the injury or complication, free of charge, as a public patient in any Australian public hospital.

17 Who is organising and funding the research?

This research project is being led by the Coordinating Principal Investigator, Professor Richard Kanaan and has been funded by the National Health and Medical Research Council (NHMRC). No member of the research team will receive a personal financial benefit from your involvement in this research project (other than their ordinary wages).

If knowledge acquired through this research project leads to discoveries that are of commercial value to the involved Universities, the study doctors or their institutions, there will be no financial benefit to you or your family from these discoveries.

18 Who has reviewed the research project?

All research in Australia and New Zealand involving humans is reviewed by an independent group of people called a Human Research Ethics Committee (HREC). The ethical aspects of this research project have been approved by the HREC of Austin Health, on behalf of the other Victorian hospital sites. Local approval to conduct the study at *[clinical care site]* has been also been granted by *[institution]*.

This project will be carried out according to the *National Statement on Ethical Conduct in Human Research (2007)*. This statement has been developed to protect the interests of people who agree to participate in human research studies.

19 Further information and who to contact

The person you may need to contact will depend on the nature of your query.

If you want any further information concerning this project or if you have any medical problems which may be related to your involvement in the project (for example, any side effects), you can contact the principal study doctor on (03) 9496 3351 or any of the following people:

Clinical contact person

Name	*[Name]*
Position	*[Position]*
Telephone	*[Phone number]*
Email	*[Email address]*

If you have any complaints about any aspect of the project, the way it is being conducted or any questions about being a research participant in general, then you may contact:

Reviewing HREC approving this research and HREC Executive Officer details

Reviewing HREC name	Austin Health Human Research Ethics Committee
HREC Executive Officer	Manager, Discovery & Innovation Unit
Telephone	(03) 9496 4090
Email	research@victri.org.au

Local HREC Office contact (Single Site - Research Governance Officer)

Name	*[Name]*
Position	*[Position]*
Telephone	*[Phone number]*
Email	*[Email address]*

Consent Form - *Adult providing own consent*

**Title**	Breathing Control Training (BCT) as a Treatment for Functional Seizures (FS)
**Short Title**	BREATHS trial
**Protocol Number (Version)**	1 (5)
**Project Sponsor**	The University of Melbourne
**Coordinating Principal Investigator/ Principal Investigator**	Professor Richard Kanaan
**Associate Investigator(s)** *(if required by institution)*	*[Associate Investigator(s)]*
**Location** *(where CPI/PI will recruit)*	*[Location where the research will be conducted]*

Consent Agreement

I have read the Participant Information Sheet, or someone has read it to me in a language that I understand.

I understand the purposes, procedures and risks of the research described in the project.

I understand that my medical record held at this site may be accessed to obtain data relevant to this research project.

I give permission for my doctors, other health professionals, hospitals or laboratories outside this hospital to release information to *[Name of Institution]* concerning my condition and treatment for the purposes of this project. I understand that such information will remain confidential.

I understand that my treatment sessions may be audio recorded for training and supervision purposes.

I have had an opportunity to ask questions and I am satisfied with the answers I have received.

I freely agree to participate in this research project as described and understand that I am free to withdraw at any time during the study without affecting my future health care.

OPTIONAL: I give permission for non-identifiable data collected from me during this project to be used for future research related to this project, or the same general area of research.

Yes ☐ No ☐ Participant Initials:________________ Date:_________________

OPTIONAL: I give permission for the researchers to retrieve data regarding my health service use from The Centre for Victorian Data Linkage (CVDL).

Yes ☐ No ☐ Participant Initials:________________ Date:_________________

I understand that, if I decide to discontinue the research project prematurely, I may be asked to attend a brief follow-up visit (phone/in person) to allow collection of information regarding my health status. However, I understand this is not mandatory.

I understand that I will be given a signed copy of this document to keep.

Declaration by Participant – for participants who have read the information

Name of Participant (please print) ______________________
Signature ______________________ Date ______________________

Declaration - for participants unable to read the information and consent form
Witness to the informed consent process<br>Name (please print) ______________________
Signature ______________________ Date ______________________<br>* Witness is not to be the Investigator, a member of the study team or their delegate. Witness must be 18 years or older.

For participants who are 16-17 years of age, a parent/guardian is required to co-sign

Declaration by Parent/Guardian – for Parent/Guardian who has read the information

Name of Child (please print) ______________________
Name of Parent/Guardian (please print) ______________________
Signature of Parent/Guardian ______________________ Date______________________

Declaration - for Parent/Guardian unable to read the information and consent form<br>See Note for Guidance on Good Clinical Practice CPMP/ICH/135/95 Section 4.8.9. A legally acceptable representative may be a witness*.
Witness to the informed consent process<br>Name (please print) ______________________
Signature ______________________ Date ______________________<br>* Witness is not to be the Investigator, member of the study team or their delegate. Witness must be 18 years or older.

Declaration by Study Doctor/Senior Researcher†

I have given a verbal explanation of the research project, its procedures and risks and I believe that the participant has understood that explanation.

Name of Study Doctor/<br>Senior Researcher† (please print) ______________________
Signature ______________________ Date ______________________

† A senior member of the research team must provide the explanation of, and information concerning, the research project.

Note: All parties signing the consent section must date their own signature.

Consent via telehealth or telephone (if applicable)

- ❒ Consent was obtained using telehealth with *[Name of Participant]* whose photographic identification was sighted by the Investigator who observed the Participant's signature being written
- ❒ Consent was obtained via telephone with *[Name of Participant]* on [DD/MMM/YYYY].
- ❒ Participant's signed consent form received by the Investigator on [DD/MMM/YYYY].
- ❒ Consent was obtained using telehealth with *[Name of Investigator]* whose photographic identification was sighted by the Participant who observed the Investigator's signature being written
- ❒ Consent was obtained via telephone with *[Name of Investigator]* on [DD/MMM/YYYY].
- ❒ Discussed with *[Participant]* via telephone on *[insert date]* and received signed consent form on *[insert date]*. Signed by *[Investigator]*.

Form for Withdrawal of Participation - *Adult providing own consent*

**Title**	Breathing Control Training (BCT) as a Treatment for Functional Seizures (FS)
**Short Title**	BREATHS trial
**Protocol Number (Version)**	1 (5)
**Project Sponsor**	The University of Melbourne
**Coordinating Principal Investigator/ Principal Investigator**	Professor Richard Kanaan
**Associate Investigator(s)** *(if required by institution)*	*[Associate Investigator(s)]*
**Location** *(where CPI/PI will recruit)*	*[Location where the research will be conducted]*

Declaration by Participant

I wish to withdraw from participation in the above research project and understand that such withdrawal will not affect my routine treatment, my relationship with those treating me or my relationship with *[Institution]*.

I permit the researchers to use data that has already been collected from me and include it in the analyses and results of this project ☐ YES ☐ NO

Name of Participant (please print) ______________________

Signature ______________________ Date ______________________

In the event that the participant's decision to withdraw is communicated verbally, the Study Doctor/Senior Researcher will need to provide a description of the circumstances below.

Declaration by Study Doctor/Senior Researcher†

I have given a verbal explanation of the implications of withdrawal from the research project and I believe that the participant has understood that explanation.

Name of Study Doctor/
Senior Researcher† (please print) ______________________

Signature ______________________ Date ______________________

† A senior member of the research team must provide the explanation of and information concerning withdrawal from the research project.

Note: All parties signing the consent section must date their own signature.
