# Supplementary material for: Breathing control training as a treatment for functional seizures (BREATHS trial): a multicentre, assessor-blinded, randomised controlled efficacy and acceptability trial study protocol
Source: BMJ Open. 2026 Jan 30;16(1):e107687. doi: 10.1136/bmjopen-2025-107687 (PMC12863316; doi:10.1136/bmjopen-2025-107687)
Supplement: Supplementary file 2 [file bmjopen-2025-107687.reviewer_comments.pdf]

PEER REVIEW HISTORY

BMJ Open publishes all reviews undertaken for accepted manuscripts. Reviewers are asked to complete a checklist review form and are provided with free text boxes to elaborate on their assessment. These free text comments are reproduced below.

ARTICLE DETAILS

Title (Provisional)

Breathing Control Training as a Treatment for Functional Seizures (BREATHS trial): A multi-centre, assessor-blinded, randomised controlled efficacy and acceptability trial study protocol

Authors

Kanaan, Richard; Duncan, Rod; Mihalopoulos, Cathrine; Braat, Sabine; O'Brien, Terence J; Velakoulis, Dennis; Kwan, Patrick; Mulder, Roger T; Cook, Mark; Mullen, Saul; Mayne, Deeanne; Oliver, Gina; Eleftheriadis, Dina; Ameen, Ozayr; Chatterton, Mary Lou; D'Souza, Wendyl; Nicolo, John-Paul; Perucca, Piero; Winton-Brown, Toby; Zaloumis, Sophie; Berlowitz, David

---

VERSION 1 - REVIEW

**Reviewer**	**1**
**Name**	**Ong, Cheung-Ter**
**Affiliation**	**Ditmanson Medical Foundation Chia-Yi Christian Hospital, Neurology**
**Date**	**19-Aug-2025**
**COI**	**None**

A multi-center, assessor-blinded, individually-randomized superiority study protocol. Hypothesis of the study is that hyperventilation may induce functional seizures and breathing control training may improve hyperventilation and reduce functional seizure. Pilot study results showed that 7 out of 10 patients who received breathing control training study improved seizure frequency, while 3 did not. The protocol aim to confirm the results. The study hypothesis, methods, and analysis are acceptable. There are, however, some points that need to be clarified.

1. There are many abbreviations that are not defined at first appearance.

2. In the pilot trial the outcome of BCT is defined as improved seizure frequency 3-4 months later. Why in the study, the outcome is defined as seizure remission (FS frequency is zero in the preceding 4 weeks) at 12 weeks after initial treatment? Why is the outcome defined as seizure remission (12 weeks after the initial treatment)?

How many patients achieved FS remission in the pilot study?

3. In the participant safety and withdrawal.

The withdrawal criteria (Emergence of epileptic seizures) need to be clearly definite.

Who confirms the emergence of epileptic seizures?

4. The dates of the study should be included in the manuscript.

5. Inclusion criteria (Self-reported FS frequency of at least 2 per month).

Was it in the past 1 year or in the preceding 4 weeks?

**Reviewer**	**2**
**Name**	**Quigg, Mark**
**Affiliation**	**University of Virginia, Neuro**
**Date**	**06-Oct-2025**
**COI**	**None**

This is a description of a planned definitive RCT for a breathing control therapy (BCT) intervention for FS. The investigators have preliminary data of an open-label study. The target group is adults with FS. The control therapy is "Befriending", defined in the MS. The primary outcome is seizure remission. A battery of secondary outcomes includes comorbidities, function/QOL, safety, acceptability, and potential biomarkers to predict response. Exploratory outcomes are measurements of trauma/significant life events and dissociation. The power estimate is designed to demonstrate 2x the rate of seizure remission seen in standard of care CBT. It is novel and will be useful to the medical community. It is well-written and well-researched. My compliments.

1. My concerns center on the relationships among the sites/timing of inpatient/EMU diagnosis of FS, testing to be performed as an inpatient vs outpatient, and the timing of recruitment, consent, and enrollment. I was expecting recruitment and consent to take place as an inpatient as video-EEG monitoring was about to begin, with subsequent enrollment designed to exclude those with epilepsy and retain those with FS.

a-Section "Recruitment" states that recruitment and enrollment will take place at their clinical care site (outpatient). At the time of diagnosis as an impatient is implied in the "Consent" section. Please be more specific when and where recruitment, consent, and enrollment will occur.

b-About 20-30% of those with FS remit soon after video-EEG after the "therapeutic explanation". Is the seizure frequency in inclusion criteria Table 1 prediagnosis baseline or

post diagnosis? If pre-diagnosis, how will the change score and seizure remission be handled for those whose seizures are tentatively remitted at the time of recruitment? If post-diagnosis, the trial is only recruiting the "refractory" group with continuing FS, a tough crowd.

c-Is there a time range after diagnosis that effects eligibility? Those with continuing seizures long after diagnosis of FS may be a population enhanced in unexpected ways (borderline personality, Munchausen, continuing abuse, …). I worry that generalizability of the findings will be limited with a "free-range" outpatient/post-diagnosis design.

d-Are all patients in the trial centers routinely monitored for tcCO2 during vEEG? If not, how is the extra testing retroactively applied? Is absence of such extra testing an exclusion?

e-I find the psychological testing battery voluminous, and my experience with FS patients makes me wonder how successful this battery can be. Are incomplete batteries "drop-outs" or screen failures? If one enrolls as an inpatient, supervised (and bored) subjects are more likely to complete the battery.

f-the outpatient recruitment strategy may cloud the designation of a "time zero", which I argue is most cleanly as the time of diagnosis of FS. Please discuss the rationale in the manuscript.

g-please pre-specify a "drop-out" in terms of the primary outcome. I think the minimum criterion for the primary outcome would be intact f/u, seizure diary, and treatment adherence through the planned 12 weeks. Also, please specify whether this an intent-to-treat paradigm.

2. Minor exclusion criteria questions (although I anticipate that things washing out in randomization will be a quick and appropriate answer):

A-In the US, some patients seek second or more opinions, which again changes the recruited population. Should there be an exclusion for two or more diagnostic admissions?

B-Would useful exclusion criteria include asthma? Borderline personality disorder?

C-Are continuing antiseizure medications a useful criterion?

VERSION 1 - AUTHOR RESPONSE

Reviewer 1:
Dr. Cheung-Ter Ong, Ditmanson Medical Foundation Chia-Yi Christian Hospital
Comments to the Author:

Comment 1 There are many abbreviations that are not defined at first appearance.

> Response
> We thank the reviewer for this helpful observation. We have carefully reviewed the manuscript and ensured that all abbreviations are defined at their first appearance in the text.

Comment 2 In the pilot trial the outcome of BCT is defined as improved seizure frequency 3-4 months later. Why in the study, the outcome is defined as seizure remission (FS frequency is zero in the preceding 4 weeks) at 12 weeks after initial treatment? Why is the outcome defined as seizure remission (12 weeks after the initial treatment)? How many patients achieved FS remission in the pilot study?

<u>Response</u>
We thank the reviewer for this insightful comment and acknowledge the change in the outcome measure from seizure improvement in the pilot study to seizure remission in the randomised trial. During the early discussions of trial design, the investigator group agreed that seizure remission would serve as a more objective and clinically meaningful outcome than reduced seizure frequency, which can be difficult to measure accurately (e.g., recall bias, idiosyncratic experience of FS). Further to this, the team reasoned that complete cessation of seizures would have a more substantial and direct impact on a patient's quality of life compared to partial improvement or reduction in seizure frequency.

In the pilot study, three of 8 for whom follow-up data were available had achieved seizure remission (defined as zero seizures during the preceding 4 weeks), though there was evidence from outside the trial that two others had also achieved remission.

Comment 3 In the participant safety and withdrawal. The withdrawal criteria (Emergence of epileptic seizures) need to be clearly definite. Who confirms the emergence of epileptic seizures?

<u>Response</u>
We agree the withdrawal criteria, emergence of epileptic seizures, needs to be defined and we have added a paragraph regarding this, in the "Participant safety and withdrawal" section.

The section on page 14 now includes this additional paragraph:

"*If a participant self-reports to the study team (e.g., during a routine study visit), or if a member of the study team identifies in the patient medical records a new diagnosis of epilepsy or the emergence of epileptic seizures, the participant will be withdrawn from the study. The diagnosis must be confirmed by an epileptologist. The participant will then be notified of their withdrawal from the trial and the reason for it. The standard withdrawal of consent procedure described above will be followed.*"

Comment 4 The dates of the study should be included in the manuscript.

<u>Response</u>
We thank the reviewer for this helpful suggestion. We have included study dates in the *Recruitment* section (page 13, line 24). "*Recruitment commenced in February 2024 is expected to remain open for a period of 4 years*"

Comment 5 Inclusion criteria (Self-reported FS frequency of at least 2 per month). Was it in the past 1 year or in the preceding 4 weeks?

<u>Response</u>

We have clarified the Inclusion criteria (page 9, line 13-15) Self-reported FS frequency of at least 2 per month in the preceding 4 weeks.

Reviewer 2: Dr. Mark Quigg, University of Virginia

Comments to the Author:
This is a description of a planned definitive RCT for a breathing control therapy (BCT) intervention for FS. The investigators have preliminary data of an open-label study. The target group is adults with FS. The control therapy is “Befriending”, defined in the MS. The primary outcome is seizure remission. A battery of secondary outcomes includes comorbidities, function/QOL, safety, acceptability, and potential biomarkers to predict response. Exploratory outcomes are measurements of trauma/significant life events and dissociation. The power estimate is designed to demonstrate 2x the rate of seizure remission seen in standard of care CBT. It is novel and will be useful to the medical community. It is well-written and well-researched. My compliments.

Comment 1

My concerns center on the relationships among the sites/timing of inpatient/EMU diagnosis of FS, testing to be performed as an inpatient vs outpatient, and the timing of recruitment, consent, and enrollment. I was expecting recruitment and consent to take place as an inpatient as video-EEG monitoring was about to begin, with subsequent enrollment designed to exclude those with epilepsy and retain those with FS.

Response
We thank the reviewer for their detailed review, positive comments on the usefulness of this study, and their thoughtful and constructive feedback that have helped strengthen the manuscript.

Comment 1 a. Section “Recruitment” states that recruitment and enrollment will take place at their clinical care site (outpatient). At the time of diagnosis as an impatient is implied in the “Consent” section. Please be more specific when and where recruitment, consent, and enrollment will occur.

Response
We thank the reviewer for their comment and acknowledge the manuscript could be more specific regarding when and where recruitment, consent, and enrolment will occur. Recruitment, consent, and enrolment may take place either in the inpatient or outpatient setting. This approach was chosen to maintain flexibility in recruitment and to accommodate participant preference and convenience. The timing is therefore flexible to accommodate the variety of circumstances in which recruitment may occur, the commonality being that the referral will be made by their clinicians after diagnosis is confirmed. Additionally, some participants will have had access to ambulatory VEEG, which would lead to recruitment at their outpatient follow-up. In the *Recruitment* section we have amended the sentence (page 13, line 24-27) to now read “*Participants will be recruited from their clinical care site (Austin, Alfred, RMH, SVHM or Christchurch), either as an inpatient or outpatient, following their diagnosis of FS*.”

In the *Consent* section (page 23, line 11) we have removed the wording “at the hospital” to avoid potential confusion.

We have also replaced the term “PICF”, which is the Australian term for consent form, with the more international “consent form” throughout, which we hope will clarify the process.

Comment 1 b About 20-30% of those with FS remit soon after video-EEG after the “therapeutic explanation”. Is the seizure frequency in inclusion criteria Table 1 prediagnosis baseline or post diagnosis? If pre-diagnosis, how will the change score and seizure remission be handled for those whose seizures are tentatively remitted at the time of recruitment? If post-diagnosis, the trial is only recruiting the “refractory” group with continuing FS, a tough crowd.

<u>Response</u>

The seizure frequency specified in the inclusion criteria is collected after diagnosis; however, it may overlap with the patient’s experience of FS prior to diagnosis, depending on how quickly we are able to recruit participants once they are diagnosed. We acknowledge that due to the occasionally protracted process of recruitment it may lead to a relatively refractory population in study cohort.

Comment 1 c Is there a time range after diagnosis that effects eligibility? Those with continuing seizures long after diagnosis of FS may be a population enhanced in unexpected ways (borderline personality, Munchausen, continuing abuse, …). I worry that generalizability of the findings will be limited with a “free-range” outpatient/post-diagnosis design.

<u>Response</u>

While we have not specified a time after diagnosis, our process has been to contact people who were diagnosed within the last year As we do not imagine this therapy would be exclusively available to the newly diagnosed, we think this range, and the more ‘chronic’ features involved, will therefore serve to enhance the generalisability rather than limit it.

Comment 1d Are all patients in the trial centers routinely monitored for tcCO2 during vEEG? If not, how is the extra testing retroactively applied? Is absence of such extra testing an exclusion?

<u>Response</u>

The plan was that all trial centers would routinely monitor tcCO2 during VEEG; in practice, this has been complex to set up, with the different centers being at different stages in set up, and is challenging to conduct even when set up. We retain it in our protocol, though many subjects will not have it, it will not be obtained retroactively in those who do not, and it will not affect inclusion, but can obviously only be analysed in those who have had it. We have amended the text in *Study objectives* section (page 06, line 30-32) to make this clearer. The section now reads:

“*In addition, for participants with routinely monitored tcCO2 during video EEG, we will examine if a respiratory biomarker of hyperventilation, tcCO2, will predict treatment response to BCT*.”

Comment 1e I find the psychological testing battery voluminous, and my experience with FS

patients makes me wonder how successful this battery can be. Are incomplete batteries "drop-outs" or screen failures? If one enrolls as an inpatient, supervised (and bored) subjects are more likely to complete the battery.

<u>Response</u>

We appreciate the reviewer's concern regarding the potential burden of the testing battery. Much thought went into the number of measures we included in the battery, including piloting with patients and we believe the duration (~40mins) strikes a good balance. Participants are also able to complete the survey over several sessions, should they need breaks. Incomplete responses are nonetheless likely but will be included in the analyses, provided that sufficient data are available to allow accurate scoring of the measure. Screen failures will not be included.

Comment 1f the outpatient recruitment strategy may cloud the designation of a "time zero", which I argue is most cleanly as the time of diagnosis of FS. Please discuss the rationale in the manuscript.

<u>Response</u>

We have added the following paragraph to the recruitment section (page 13, line 50):

"*Recruitment from both inpatient and outpatient sources may lead to recruitment of a more chronic, and thus possibly more refractory, cohort, but will increase the number of potential recruits and enhance generalisability*."

Comment 1g please pre-specify a "drop-out" in terms of the primary outcome. I think the minimum criterion for the primary outcome would be intact f/u, seizure diary, and treatment adherence through the planned 12 weeks. Also, please specify whether this an intent-to-treat paradigm.

<u>Response</u>

We thank the reviewer for this thoughtful suggestion. We have decided not to pre-specify a dropout rate in the study design because of the unpredictable nature of the population and because intention-to-treat analysis will be used.

We have included the sentence "*All analyses will follow the intention-to-treat basis principle*." In *Statistical analysis* section (page 21, lines 30-32)

Comment 2. Minor exclusion criteria questions (although I anticipate that things washing out in randomization will be a quick and appropriate answer):

Comment 2a -In the US, some patients seek second or more opinions, which again changes the recruited population. Should there be an exclusion for two or more diagnostic admissions?

<u>Response</u>

This is an important consideration, and repeat admissions definitely occur, however in Australia and New Zealand, diagnostic admissions will be almost entirely to public hospitals, and thus less at the patient's discretion, and largely because the first admission did not capture an event. Though it will therefore introduce a bias, it is perhaps a different one than in the US, and we will rely, as the reviewer suggests, on randomisation to take care of it.

Comment 2b-Would useful exclusion criteria include asthma? Borderline personality disorder?

Response

We did not consider borderline personality disorder as an exclusion, though we anticipate that excluding it would have improved the efficacy of the intervention. We have been motivated in designing our criteria to maximise recruitment potential and generalisability. Asthma however was something we considered at some length; again motivated by recruitment potential and generalisability, we decided to keep it in, but are specifically 'measuring' it by clinical questions in our assessment so that we may accommodate it in the analysis.

Comment 2c-Are continuing antiseizure medications a useful criterion?

Response

This is undoubtedly an important consideration, but one where we felt we had to rely on randomisation to address.

We trust we have addressed your concerns and look forward to hearing from you in due course

---

VERSION 2 - REVIEW

**Reviewer**	**2**
**Name**	**Quigg, Mark**
**Affiliation**	**University of Virginia, Neuro**
**Date**	**18-Dec-2025**
**COI**	

The authors answered my questions. I still consider that the patients are enrolled ona variable schedule following diagnosis for the reasons given in my first review, but I also understand that enrollment pressures have lead to the current design.
